# Evaluation of an AI-assisted digital health follow-up system integrating humanistic care for patients undergoing chemotherapy: a prospective quasi-experimental study

**DOI:** 10.3389/fpubh.2026.1863027

**Published:** 2026-08-12

**Authors:** Luning Jing, Baoling Ye, Shanshan Liu

**Affiliations:** 1 The Department of Radiation Oncology, Shinan Campus, Affiliated Hospital of Qingdao University, Qingdao, China; 2 The Department of Gynecology, Pingdu Campus, Affiliated Hospital of Qingdao University, Qingdao, China

**Keywords:** artificial intelligence, chemotherapy, digital health, humanistic care, oncology nursing, patient-reported outcomes

## Abstract

**Background:**

Chemotherapy imposes substantial physical and psychological burdens that may reduce treatment adherence and quality of life. Traditional follow-up is often fragmented, reactive, and insufficiently personalized. Artificial intelligence (AI) may improve continuity, responsiveness, and patient-centered care; however, its integration with humanistic care remains underexplored.

**Methods:**

This prospective, single-center, quasi-experimental study was conducted from January to June 2024. A total of 189 patients undergoing chemotherapy were allocated to an AI-assisted follow-up group (*n* = 96) or a conventional care group (*n* = 93). The nurse-supervised AI-assisted follow-up system integrated rule-based risk monitoring, DeepSeek-based large language model-assisted communication, personalized follow-up, and data visualization. Outcomes included self-management ability, quality of life (QoL), and patient satisfaction. Between-group comparisons used independent-samples *t*-tests, with adjusted analyses performed using analysis of covariance.

**Results:**

At 3 months, the AI-assisted group had higher self-management ability scores (168.83 ± 7.03 vs. 150.11 ± 7.39), QoL scores (66.34 ± 4.30 vs. 59.81 ± 4.46), and satisfaction scores (52.35 ± 2.44 vs. 48.75 ± 2.48) than the conventional care group (all *P* < 0.001). Follow-up completion was 96%, and the 24-h response rate was 98%. Of 214 generated alerts, 87.4% were clinically validated.

**Conclusion:**

The nurse-supervised AI-assisted follow-up system was feasible and associated with better short-term patient-reported outcomes than conventional follow-up. Its distinguishing feature was the integration of rule-based risk management, large language model-assisted communication, and operationalized humanistic care within a closed-loop oncology nursing workflow. Randomized multicenter studies are required to confirm these findings.

## 1 Introduction

Cancer remains a leading cause of morbidity and mortality (1). Although chemotherapy is essential to comprehensive cancer care, it often causes fatigue, pain, nausea, immunosuppression, and anxiety (2, 3). Patients may receive fragmented information, insufficient symptom management, and limited post-discharge professional support. This can reduce adherence to therapy and have a negative effect on quality of life (QoL) (4, 5). Building a comprehensive, patient-centered follow-up system that provides continuous support is therefore critical to optimal nursing care. Traditional oncology follow-up (periodic visits/telephone calls) is constrained by delayed communication, limited individualization, and poor capacity to detect evolving needs (6). In contrast, contemporary nursing philosophy emphasizes holistic, continuous care that integrates physiological, psychological, and social dimensions (7). Advances in artificial intelligence (AI) and digital health create opportunities to transform follow-up from reactive to proactive, data-assisted, and emotionally supportive care (8–10). Although AI-assisted follow-up systems can predict complications, personalize education, and improve efficiency, many focus narrowly on information delivery or symptom tracking, and overlook empathy and emotional support, which are central to humanistic care (11, 12).

Humanistic care is conceptually grounded in caring science, with Watson's theory of human caring representing one of its most influential theoretical foundations. Watson emphasizes that nursing is not only a technical practice but also a humanistic discipline centered on empathy, compassion, and holistic care (13, 14). According to this theory, effective nursing should address not only patients' physical conditions but also their psychological, emotional, and existential needs, thereby promoting overall wellbeing. DeepSeek, an AI-assisted follow-up system that integrates large language models with structured clinical data processing, enables real-time information analysis, personalized decision support, and interactive communication (15). Compared with conventional rule-based systems, such platforms offer enhanced capabilities in natural language understanding, patient engagement, and adaptive response generation, thereby providing a more flexible and responsive digital health framework. We therefore developed a nurse-supervised AI-assisted digital health follow-up system that combined rule-based clinical risk stratification with DeepSeek-based large language model-assisted communication and operationalized humanistic care.

Existing digital oncology follow-up and electronic patient-reported outcome systems have already incorporated functions such as symptom monitoring, automated alerts, patient education, and clinician communication. Therefore, the novelty of the present intervention does not lie in any single technological function. Rather, it lies in the integration of three elements within a unified nursing workflow: rule-based clinical risk stratification, large language model-assisted patient communication, and nurse-supervised humanistic care. Unlike systems focused primarily on data collection or automated symptom alerts, the present system linked patient-reported information to structured risk assessment, empathetic and context-sensitive communication, and timely escalation to oncology nurses. Humanistic care was operationalized as a predefined component of the intervention through empathetic language templates, emotional distress screening, individualized reassurance, and nurse-led psychosocial follow-up. This hybrid and closed-loop design was intended to preserve clinician oversight while extending continuous and emotionally responsive care beyond the hospital setting. Accordingly, this pilot study aimed to evaluate the feasibility and short-term outcome associations of a nurse-supervised, AI-assisted digital follow-up system that integrates rule-based risk management, LLM-assisted communication, and operationalized humanistic care for patients undergoing chemotherapy.

## 2 Materials and methods

### 2.1 Study design and setting

This study was designed as a prospective, single-center, quasi-experimental feasibility study conducted from January to June 2024 at the Affiliated Hospital of Qingdao University in Qingdao, China. The study pilot tested a newly developed AI-assisted digital health follow-up system that incorporated rule-based decision support combined with structured clinical logic and real-time data integration. Eligible patients were consecutively recruited from the Department of Oncology and allocated to either an AI-assisted follow-up group or a conventional care group. Because allocation was based on patient preference and clinical scheduling rather than randomization, the study was susceptible to self-selection and unmeasured confounding. The adjusted analyses were intended to reduce imbalance in the measured demographic and clinical variables but could not eliminate differences in unmeasured characteristics between the groups.

### 2.2 Participants

Patients were eligible to participate if they were aged ≥18 years, with pathologically confirmed malignancies, were receiving standard chemotherapy, and had stable vital signs. All participants were required to be capable of using a smartphone or other digital device and to provide informed consent prior to enrollment.

Patients were excluded if they had severe cognitive or psychiatric disorders that could interfere with participation, were enrolled in another digital or psychological intervention study, or had an expected survival time of less than 3 months.

### 2.3 AI-assisted follow-up system

The AI-assisted follow-up system was implemented as a nurse-supervised hybrid AI-assisted follow-up system combining rule-based clinical decision support with DeepSeek-based large language model-assisted communication. The rule-based component processed structured symptom reports and selected clinical variables using predefined thresholds and decision rules. It was responsible for symptom-based risk stratification and alert generation. Alerts were triggered when patient-reported symptoms met predefined criteria related to severity, duration, or combinations requiring further clinical attention.

The DeepSeek-based large language model was used primarily to process patient-entered free-text information and support patient communication. Its functions included generating patient-friendly explanations, empathetic responses, and standardized health education messages based on patients' reported symptoms and concerns. Standardized prompt templates were used for common scenarios, including symptom reporting, treatment-related questions, emotional distress, and self-management guidance. AI-generated content was constrained by predefined clinical rules, approved nursing education materials, and structured response templates. The large language model was not used to independently establish diagnoses, prescribe medication, modify chemotherapy regimens, or make autonomous clinical decisions.

All alerts requiring clinical attention were reviewed by trained oncology nurses. Nurses verified the patient-reported information, assessed the clinical relevance of the alert, and determined whether telephone follow-up, further assessment, outpatient review, or physician referral was required. Nurses could revise, withhold, or override AI-generated content, and final clinical responsibility remained with the healthcare professionals.

Humanistic care was defined as a structured approach incorporating empathy, respect, emotional support, individualized communication, and timely nurse involvement. It was operationalized through standardized empathetic response patterns, screening for emotional distress, individualized reassurance and education, and escalation to nursing staff when additional psychosocial or clinical support was required. Unlike routine patient-centered communication, these elements were predefined and embedded into the intervention workflow. Humanistic care was not evaluated as an independent outcome using a validated instrument; therefore, the study assessed the integrated intervention as a whole rather than the isolated effect of its humanistic care component.

Potential adverse events and unintended consequences related to the AI-assisted follow-up system were monitored throughout the intervention period. These included inappropriate or misleading AI-generated responses, technical failures, delayed nurse responses, unsuccessful message delivery, patient misunderstanding, and difficulties in system use. All clinically relevant AI-generated content and alerts were reviewed by trained oncology nurses, who could revise, withhold, or override the content when necessary.

**Figure 1:**
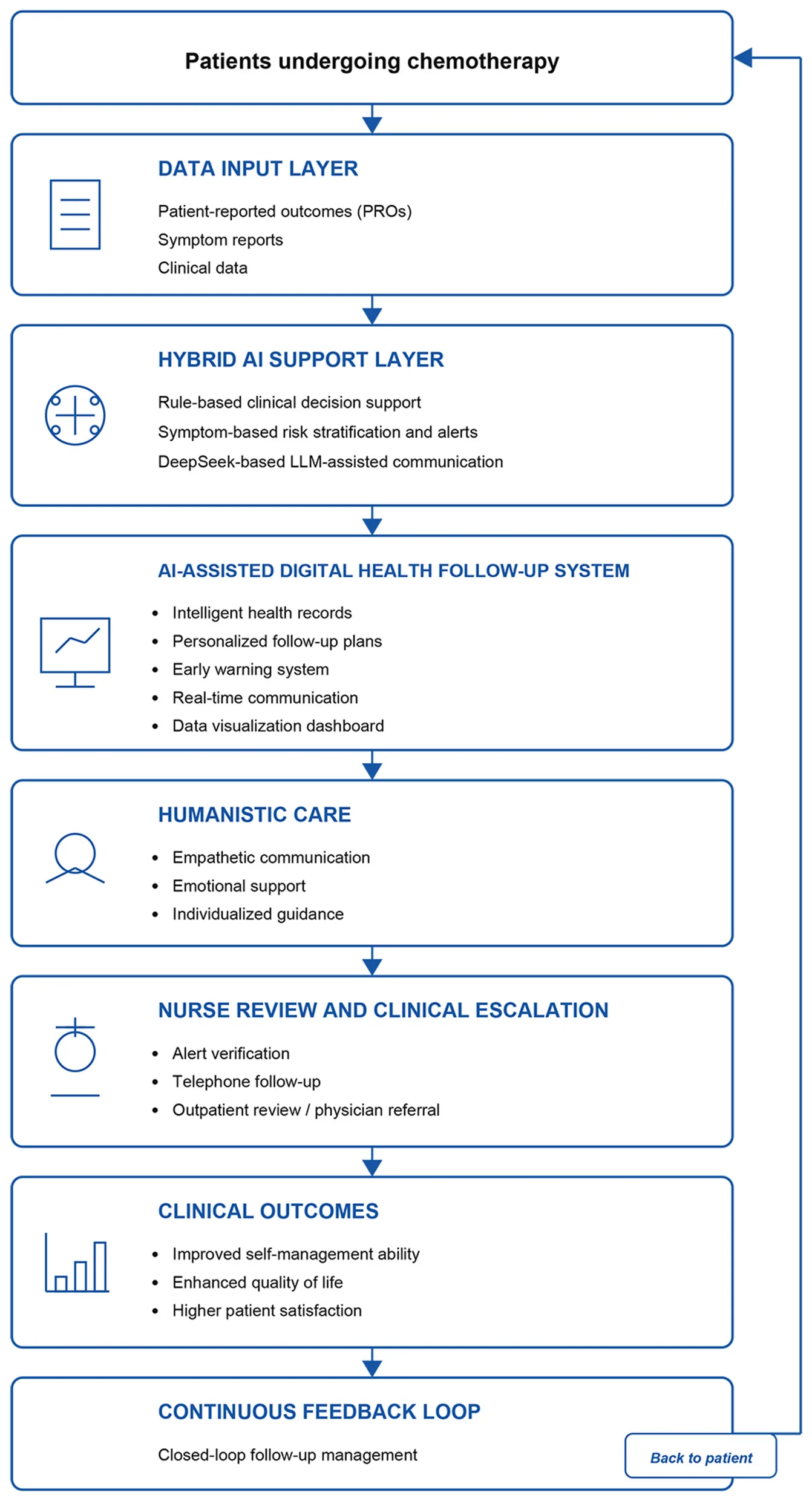
Architecture and workflow of the AI-assisted digital health follow-up system integrating humanistic care.

The overall architecture and workflow of the nurse-supervised AI-assisted digital health follow-up system, including data input, hybrid AI support, humanistic care, nurse review, clinical escalation, and continuous feedback, are illustrated in Figure 1.

### 2.4 Implementation procedures

Before study initiation, participating oncology nurses received structured training on system operation, symptom assessment, alert verification, empathetic communication, data entry, and escalation procedures. The training included standardized operating instructions, case-based demonstrations, and supervised practice. Only nurses who had completed the training participated in the intervention.

The system was integrated into the routine oncology follow-up workflow. Patient-reported symptoms and alerts were reviewed by designated nurses during scheduled working periods, and clinically relevant alerts were followed by telephone assessment, additional nursing guidance, outpatient review, or referral to a physician when necessary. Standardized follow-up procedures, response templates, and escalation criteria were used to improve intervention fidelity. The research team periodically reviewed system records and nursing responses to identify deviations from the protocol and provide corrective feedback.

During implementation, the main challenges included differences in patients' familiarity with digital devices, occasional difficulties in symptom reporting, temporary network or system-access problems, and additional nursing workload related to alert review. Important facilitators included the simple interface, structured response templates, predefined clinical rules, and direct access to nursing support. Informal feedback from participating nurses indicated that the system improved the organization of follow-up information and facilitated early identification of patient concerns, although no formal healthcare professional acceptance scale was administered.

### 2.5 Comparison group

Patients in the comparison group received conventional care, which included routine telephone follow-up every 2 weeks and outpatient visits after each chemotherapy cycle. Standard nursing guidance on medication, diet, and side-effect management was provided according to institutional protocols. No digital monitoring, automated risk alerts, or AI-assisted communication tools were used in the conventional care group.

### 2.6 Outcomes

The primary outcomes included self-management ability, QoL, and patient satisfaction. Self-management ability was assessed using a study-specific 20-item questionnaire developed before data collection based on chronic disease self-management theory, relevant oncology nursing literature, and the clinical characteristics of patients undergoing chemotherapy (16, 17). The questionnaire covered symptom management, treatment adherence, self-monitoring, and emotional and daily life management. Each item was scored from 0 to 10, yielding a total score of 0–200, with higher scores indicating better self-management ability. Its content was reviewed by clinicians and nurses involved in chemotherapy care, and internal consistency was acceptable in the present study (Cronbach's α = 0.85). QoL was assessed using the global health status/QoL scale of the EORTC QLQ-C30, scored from 0 to 100, with higher scores indicating better QoL (18). Patient satisfaction was measured using a study-specific 12-item questionnaire based on patient-centered care principles and routine oncology nursing practice (19). It covered responsiveness, communication effectiveness, perceived empathy, and overall evaluation, with each item scored from 0 to 5 and a total score of 0–60. Self-management ability and QoL were assessed at baseline and 3 months, whereas patient satisfaction was assessed at 3 months only.

The study-specific questionnaires were developed to capture chemotherapy-related self-management tasks and nursing-service experiences relevant to the intervention. However, they had not undergone comprehensive psychometric validation, including assessment of construct validity, criterion validity, responsiveness, or test–retest reliability; therefore, the corresponding findings should be considered exploratory and interpreted with caution. The full item sets and scoring methods are provided in Supplementary Tables S1, S2. Feasibility outcomes included follow-up completion, the 24-h nurse response rate, clinically validated alerts, and system usability. Follow-up completion was defined as completion of the scheduled 3-month assessment, whereas the 24-h response rate was defined as the proportion of patient inquiries or alerts that received a nurse response within 24 h. Clinically validated alerts were defined as alerts reviewed by trained oncology nurses and judged, according to predefined symptom and escalation criteria, to require further assessment or follow-up. System usability was assessed using a study-specific 5-item questionnaire covering ease of use, information clarity, response convenience, perceived usefulness, and overall satisfaction. Each item was scored from 1 to 5, and the mean item score was reported. This usability questionnaire was also used as an exploratory measure and had not undergone formal psychometric validation. As no formal feasibility threshold was prespecified, these indicators were interpreted descriptively.

### 2.7 Intervention exposure

Intervention exposure was assessed using system log data and nursing follow-up records. Exposure indicators included the number of patient–system interactions, completed symptom reports, patient inquiries, AI-generated alerts, nurse interventions following alerts, and duration of active engagement during the 3-month intervention period. A patient–system interaction was defined as a completed symptom report, follow-up response, or submitted inquiry. A nurse intervention was defined as telephone follow-up, additional nursing guidance, recommendation for outpatient assessment, or physician referral initiated after review of an AI-generated alert.

### 2.8 Statistical analysis

Statistical analyses were performed using SPSS version 26.0 (IBM Corp, Armonk, NY, USA). Normality assumptions were assessed prior to parametric testing. Normally distributed continuous variables were reported as the mean ± standard deviation and groups were compared using independent-samples *t*-tests. Categorical variables were reported as frequencies and percentages and groups were compared using χ^2^ tests. Baseline between-group comparisons were performed for outcomes assessed at baseline, including self-management ability and quality of life (QoL). To address potential confounding inherent in the quasi-experimental design, adjusted analyses were conducted using analysis of covariance (ANCOVA). For self-management ability and QoL, the corresponding baseline score, together with age, sex, cancer type, number of chemotherapy cycles, and comorbidities, was included as covariates. For patient satisfaction, which was assessed only at 3 months, adjusted analyses included age, sex, cancer type, number of chemotherapy cycles, and comorbidities as covariates. Adjusted mean differences were reported with 95% confidence intervals (CIs) and *P-values* to enhance interpretability. Sensitivity analyses were performed for self-management ability and QoL using change-from-baseline scores to assess the robustness of the findings and whether the adjusted results were consistent with those of the unadjusted analyses. Standardized effect sizes for the between-group comparisons at 3 months were calculated using Cohen's *d*, defined as the difference between group means divided by the pooled standard deviation. Values of approximately 0.2, 0.5, and 0.8 were interpreted as small, moderate, and large effects, respectively. Cohen's *d* was based on the unadjusted 3-month group means and standard deviations and was reported alongside the adjusted mean differences, 95% confidence intervals, and *P-values*. Data completeness was assessed before analysis. As all participants included in the final analysis had complete baseline and 3-month outcome data, no statistical imputation was required. Given the non-randomized allocation, the adjusted analyses accounted only for measured covariates. Potentially relevant unmeasured characteristics, including digital literacy, educational level, motivation, socioeconomic status, health engagement, and previous experience with digital technologies, were not available for adjustment. Therefore, the findings were interpreted as adjusted associations rather than causal effects.

### 2.9 Ethics, data protection, and AI safety

This study was approved by the Ethics Committee of the Affiliated Hospital of Qingdao University (QYFTWZLL29857), and all participants provided written informed consent. Because the study evaluated a digital nursing intervention without introducing new therapeutic procedures, formal clinical trial registration was not required. Patient data were de-identified and stored on secure hospital servers, with direct identifiers kept separately and access limited to authorized personnel. The DeepSeek-based large language model was deployed within the hospital's controlled information environment, and patient data were processed locally without transmission to external commercial servers. AI-generated content was limited to communication, health education, and follow-up support and was not used for independent diagnosis, prescribing, treatment modification, or autonomous clinical decision-making. Standardized prompts, predefined clinical rules, approved educational content, and escalation criteria were used to reduce inappropriate recommendations. AI-generated responses and clinically important alerts were reviewed by trained oncology nurses, who could revise, withhold, or override the content, while final clinical responsibility remained with the healthcare professionals.

## 3 Results

### 3.1 Baseline characteristics and baseline outcome measures

A total of 189 patients were included in the final analysis, including 96 in the AI-assisted follow-up group and 93 in the conventional care group. At baseline, the two groups had similar self-management ability scores (142.08 ± 7.42 vs. 140.10 ± 7.39) and QoL scores (57.66 ± 4.63 vs. 56.81 ± 4.46). Patient satisfaction was not assessed at baseline. No significant between-group differences were observed in demographic or clinical characteristics, including age, sex, cancer type, number of chemotherapy cycles, and comorbidities (Table 1). All participants completed the 3-month outcome assessments, with no withdrawals, missing follow-up assessments, or incomplete questionnaires. Therefore, all 189 patients were included in the final analyses, and no data imputation was required.

**Table 1:** Baseline demographic and clinical characteristics of participants according to group.

Variable	AI-assisted follow-up group (*n* = 96)	Conventional care group (*n* = 93)	*t/χ^2^* value	*P-value*
Age (years), mean ± SD	55.4 ± 10.3	54.9 ± 9.8	0.31	0.758
Sex, *n* (%)			0.03	0.867
Male	48	45		
Female	48	48		
Cancer type, *n* (%)			0.26	0.967
Lung	27	25		
Breast	25	22		
Colorectal	30	31		
Others	14	15		
No. of chemotherapy cycles, mean ± SD	4.10 ± 1.30	4.00 ± 1.20	0.48	0.631
Patients with comorbidities, *n* (%)	37 (38.5%)	35 (37.6%)	0.01	0.918

### 3.2 Self-management ability

At baseline, self-management ability scores were similar between the AI-assisted follow-up group and the conventional care group (142.08 ± 7.42 vs. 140.10 ± 7.39). At 3 months, the AI-assisted follow-up group had higher scores than the conventional care group (168.83 ± 7.03 vs. 150.11 ± 7.39; Table 2). After adjustment for baseline scores and prespecified covariates, the between-group difference remained statistically significant (adjusted mean difference 16.91, 95% CI 16.57 to 17.25, *P* < 0.001). The standardized effect size was large (Cohen's *d* = 2.60). Sensitivity analysis using change-from-baseline scores produced results consistent with the main adjusted analysis.

**Table 2:** Baseline, 3-month, and adjusted between-group comparisons of primary outcomes.

Outcome	AI-assisted follow-up group (*n* = 96) Baseline	Conventional care group (*n* = 93) Baseline	AI-assisted follow-up group (*n* = 96) 3 months	Conventional care group (*n* = 93) 3 months	Adjusted mean difference (95% CI)	Cohen's *d*	*P-value*
Self-management ability	142.08 ± 7.42	140.10 ± 7.39	168.83 ± 7.03	150.11 ± 7.39	16.91 (16.57 to 17.25)	2.60	< 0.001
QoL (EORTC QLQ-C30 global health status/QoL scale)	57.66 ± 4.63	56.81 ± 4.46	66.34 ± 4.30	59.81 ± 4.46	5.88 (5.71 to 6.05)	1.49	< 0.001
Patient satisfaction^*^	—	—	52.35 ± 2.44	48.75 ± 2.48	3.85 (3.72 to 3.99)	1.46	< 0.001

Data are presented as mean ± standard deviation. For self-management ability and quality of life (QoL), adjusted between-group comparisons at 3 months were performed using analysis of covariance (ANCOVA), with the corresponding baseline score, age, sex, cancer type, number of chemotherapy cycles, and comorbidity included as covariates. For patient satisfaction, adjusted analysis included age, sex, cancer type, number of chemotherapy cycles, and comorbidity as covariates; no baseline satisfaction assessment was available. QoL, quality of life; CI, confidence interval. Cohen's d was calculated from the unadjusted 3-month group means and pooled standard deviations and represents the standardized magnitude of the between-group difference.

^*^Patient satisfaction was assessed at 3 months only.

### 3.3 Quality of life

At baseline, QoL scores were similar between the AI-assisted follow-up group and the conventional care group (57.66 ± 4.63 vs. 56.81 ± 4.46). At 3 months, the AI-assisted follow-up group had higher QoL scores than the conventional care group (66.34 ± 4.30 vs. 59.81 ± 4.46; Table 2). After adjustment for baseline scores and prespecified covariates, the between-group difference remained statistically significant (adjusted mean difference 5.88, 95% CI 5.71 to 6.05, *P* < 0.001). The standardized effect size was large (Cohen's *d* = 1.49).

### 3.4 Patient satisfaction

Patient satisfaction was assessed at 3 months only. The AI-assisted follow-up group had higher patient satisfaction scores than the conventional care group (52.35 ± 2.44 vs. 48.75 ± 2.48; Table 2). After adjustment for prespecified covariates, the between-group difference remained statistically significant (adjusted mean difference 3.85, 95% CI 3.72 to 3.99, *P* < 0.001). The standardized effect size was large (Cohen's *d* = 1.46).

### 3.5 Feasibility and engagement

During the 3-month intervention period, 92 of 96 participants in the AI-assisted follow-up group completed the scheduled follow-up assessment, corresponding to a completion rate of 96%. A nurse response was provided within 24 h for 94 of 96 recorded inquiries or alerts (98%). The system generated 214 alerts, of which 187 (87.4%) were reviewed by trained oncology nurses and judged to require further assessment or follow-up according to the predefined symptom and escalation criteria. The mean usability score was 4.6 ± 0.5 on the study-specific 5-item questionnaire. As no formal feasibility threshold was prespecified, these indicators were interpreted descriptively and suggested generally favorable engagement and usability during the study period (Table 3).

**Table 3:** Feasibility and engagement indicators of the AI-assisted follow-up system.

Indicator	Value
Follow-up completion rate, *n/N* (%)	92/96 (96%)
24-h response rate, *n/N* (%)	94/96 (98%)
Clinically validated AI alerts, *n/N* (%)	187/214 (87.4%)
Usability score, mean ± SD	4.6 ± 0.5
Follow-up completion rate, *n/N* (%)	92/96 (95.8%)
24-h nurse response rate, *n/N* (%)	94/96 (97.9%)
Patient–system interactions, *n*	1,248
Interactions per participant, mean ± SD	13.0 ± 3.6
Completed symptom reports, *n*	876
Symptom reports per participant, mean ± SD	9.1 ± 2.7
AI-generated alerts, *n*	214
Clinically validated alerts, *n/N* (%)	187/214 (87.4%)
Alerts resulting in nurse intervention, *n/N* (%)	168/214 (78.5%)
Duration of active engagement, median (IQR), weeks	11.4 (10.0–12.0)
Usability score, mean ± SD	4.6 ± 0.5

Follow-up completion was defined as completion of the scheduled 3-month assessment. The 24-h response rate was defined as the proportion of recorded patient inquiries or alerts receiving a nurse response within 24 h. Clinically validated alerts were AI-generated alerts reviewed by oncology nurses and judged to require additional assessment or follow-up according to predefined clinical criteria. Usability was assessed using a study-specific 5-item questionnaire scored from 1 to 5; the mean item score is reported.

No serious adverse events related to the AI-assisted follow-up system were observed during the study. No inappropriate AI-generated recommendation resulted in diagnosis, medication adjustment, treatment modification, or delayed clinical care. No major technical failure or unsuccessful message delivery affected the completion of follow-up assessments. Minor user-related difficulties, when encountered, were resolved through nurse guidance and did not result in participant withdrawal or interruption of clinical care.

### 3.6 Intervention exposure

During the 3-month intervention period, participants in the AI-assisted group completed 1,248 patient–system interactions, corresponding to a mean of 13.0 ± 3.6 interactions per participant. A total of 876 symptom reports were submitted, with a mean of 9.1 ± 2.7 reports per participant, together with 372 patient inquiries. The system generated 214 alerts, of which 187 (87.4%) were clinically validated by oncology nurses. A total of 168 alerts (78.5%) resulted in additional nurse intervention, including 96 telephone follow-ups, 51 additional nursing guidance sessions, 14 recommendations for outpatient evaluation, and 7 physician referrals. The median duration of active engagement was 11.4 weeks (interquartile range, 10.0–12.0), and 84 participants (87.5%) remained engaged for at least 10 weeks (Table 3).

## 4 Discussion

This pilot study evaluated a nurse-supervised, AI-assisted digital follow-up system for patients undergoing chemotherapy. The AI-assisted follow-up group showed significantly higher self-management ability, QoL, and patient satisfaction scores than the conventional care group, consistent with previous studies on digital health-supported oncology care (20, 21). The standardized effect sizes for all three outcomes were large, suggesting substantial between-group differences in magnitude. However, these effect sizes were based on unadjusted 3-month comparisons and should be interpreted cautiously because of the non-randomized design and the use of study-specific instruments for self-management ability and patient satisfaction. The novelty of the present system did not lie in individual functions such as symptom monitoring or automated alerts, but in integrating rule-based risk stratification, large language model-assisted communication, and operationalized humanistic care within a closed-loop, nurse-supervised workflow. This design linked patient-reported information with empathetic communication, nurse review, and timely clinical escalation while avoiding autonomous AI decision-making.

From a digital health perspective, this study contributes to the growing body of evidence that technology-enabled follow-up systems can improve patient engagement and continuity of care. Previous studies have reported that digital interventions, such as telemonitoring platforms and mobile health applications, can facilitate symptom tracking and improve communication between patients and healthcare providers (22–24). However, many existing systems focus primarily on data collection and automated alerts, with limited attention to individualized care and emotional support. In contrast, the system evaluated in this study integrates real-time monitoring, risk stratification, and nurse–patient interaction within a humanistic care framework. By combining technological functionality with empathetic, patient-centered care, this integration is an important advancement over previous systems. These findings suggest that the integration of humanistic care was not only conceptual but also operationalized through empathetic communication, emotional support, and individualized interaction within the digital system.

Several mechanisms may explain the improved outcomes in the AI-assisted follow-up group. First, the provision of personalized follow-up plans and continuous feedback likely enhanced patient engagement and adherence by strengthening self-management (25, 26). Second, real-time nurse–patient communication reduced delays in symptom management and provided timely professional support, which may have alleviated anxiety and uncertainty during chemotherapy (27, 28). Third, the risk identification and the alert system enabled early detection of potential complications, allowing for prompt intervention and preventing symptom escalation. Together, these components establish a closed-loop, responsive care model that extends beyond traditional episodic follow-up and supports both behavioral and clinical improvements (29).

The higher QoL observed in the AI-assisted follow-up group may reflect better symptom management and more responsive support during chemotherapy, consistent with previous studies showing that digital symptom monitoring and patient-reported outcome systems can improve QoL in patients with cancer (22, 23). The adjusted between-group difference in the EORTC QLQ-C30 global health status/QoL score was 5.88 points. Previous studies have shown that minimally important differences for the EORTC QLQ-C30 vary by cancer type, scale, direction of change, and analytical context, with some group-level estimates ranging from approximately 5 to 10 points. Thus, the observed difference may represent a small but potentially clinically meaningful benefit. However, because this study included patients with different cancer types and did not prespecify a cancer-specific minimal important difference, the clinical significance should be interpreted cautiously. The higher patient satisfaction and QoL scores also suggest that effective digital follow-up may depend not only on technological functions but also on preserving responsive and humanistic care.

Similarly, the higher patient satisfaction in the AI-assisted follow-up group highlights the importance of responsiveness, personalization, and emotional support in patient-centered care. The system also improved nursing workflow efficiency by automating routine processes such as data collection and risk screening. This allowed healthcare professionals to focus more on complex clinical decision-making and psychosocial support. These findings are consistent with previous studies that have shown that AI-assisted follow-up systems can enhance clinical efficiency and support healthcare delivery (30, 31).

The findings of this study have important implications from a public health perspective. AI-assisted follow-up systems have the potential to extend healthcare services beyond hospital settings, improving accessibility and continuity of care, particularly for patients in remote or resource-limited areas (32, 33). Furthermore, scalable AI-assisted follow-up systems could help address challenges related to staffing shortages and increasing healthcare demand. As the global burden of cancer continues to rise, such innovative care models could contribute to more efficient and equitable healthcare delivery. Implementation challenges, including system integration, staff training, and data security, should also be considered in real-world settings (34, 35).

This study has several limitations. First, the quasi-experimental design and non-randomized allocation may have introduced selection bias. Although the analyses adjusted for measured baseline and clinical variables, unmeasured factors such as digital literacy, education, motivation, socioeconomic status, and previous experience with digital technologies may still have influenced the results. Therefore, the findings should be interpreted as associations rather than causal effects. Second, this was a single-center study with a relatively small sample and short follow-up period, which limits generalizability and the assessment of long-term outcomes. Because participants were required to use a smartphone or another digital device, the findings may not apply to older adults, patients with limited digital literacy, or settings with different technological resources. Third, the outcomes were mainly patient-reported, and the study-specific self-management ability and patient satisfaction questionnaires had not undergone comprehensive psychometric validation. Although humanistic care was embedded through predefined communication and escalation procedures, it was not independently measured using a validated instrument; therefore, its specific contribution cannot be separated from the overall intervention effect. In addition, intervention fidelity, implementation barriers, and healthcare professional acceptance were not assessed using standardized measures. Future multicenter randomized studies with larger and more diverse samples, longer follow-up, validated outcome measures, and formal implementation evaluation are needed to confirm these findings and further assess feasibility, safety, scalability, and cost-effectiveness (36).

## 5 Conclusion

The nurse-supervised AI-assisted digital follow-up system was feasible to implement and was associated with higher short-term self-management ability, QoL, and patient satisfaction scores than conventional follow-up among patients undergoing chemotherapy. By integrating rule-based risk stratification, large language model-assisted communication, and humanistic care within a closed-loop nursing workflow, the system may provide a practical approach to improving continuity and responsiveness of oncology follow-up. However, given the non-randomized, single-center design and limited follow-up, these findings should be interpreted cautiously and confirmed in larger multicenter randomized studies.

## Data Availability

The raw data supporting the conclusions of this article will be made available by the authors, without undue reservation.
