# Supplementary material for: Evaluation of an AI-assisted digital health follow-up system integrating humanistic care for patients undergoing chemotherapy: a prospective quasi-experimental study
Source: Front Public Health. 2026 Aug 12;14:1863027. doi: 10.3389/fpubh.2026.1863027 (PMC13506697; doi:10.3389/fpubh.2026.1863027)
Supplement: Supplementary file 1 [file Table_1.docx]

Supplementary Material 1. Full item sets and scoring methods for the study-specific self-management ability questionnaire and nursing service satisfaction questionnaire

This supplementary material provides the full item sets, domain structure, and scoring methods for the two study-specific instruments used as primary outcome measures in the present study: (1) the self-management ability questionnaire for patients undergoing chemotherapy and (2) the nursing service satisfaction questionnaire. Both instruments were developed before data collection for this study based on theoretical considerations, relevant literature, and the clinical characteristics of the target population. These instruments had not undergone formal psychometric validation prior to their use in this study.

Supplementary Table S1. Full item set of the study-specific self-management ability questionnaire

Instruction to participants: This questionnaire was used to assess self-management ability during chemotherapy. Participants were asked to rate each statement according to their actual experience over the past month on a scale from 0 (completely unable to do) to 10 (completely able to do).

| No. | Item statement | Domain |
| --- | --- | --- |
| 1 | I can actively understand and record possible chemotherapy-related symptoms, such as nausea and fatigue. | Symptom management |
| 2 | When nausea or vomiting occurs, I can effectively use the methods recommended by my doctor to relieve it. | Symptom management |
| 3 | I can accurately identify which symptoms, such as fever or severe abdominal pain, require immediate medical attention. | Symptom management |
| 4 | I can appropriately manage chemotherapy-related fatigue by balancing activity and rest. | Symptom management |
| 5 | I can adjust my diet to cope with chemotherapy-related loss of appetite or taste changes. | Symptom management |
| 6 | I can strictly take oral antitumor medications at the prescribed time and dose. | Treatment adherence |
| 7 | I can attend scheduled chemotherapy sessions on time without delaying or canceling them unnecessarily. | Treatment adherence |
| 8 | I can actively cooperate with all required examinations before and after chemotherapy, such as blood tests and liver and kidney function tests. | Treatment adherence |
| 9 | I can follow medical instructions for the daily care and observation of my catheter (e.g., PICC). | Treatment adherence |
| 10 | Even when I feel uncomfortable, I can still adhere to the treatment plan recommended by my doctor. | Treatment adherence |
| 11 | I can measure and record my body temperature and body weight every day at regular times. | Self-monitoring |
| 12 | I can actively observe whether there are any bleeding spots or ulcers on my skin or oral mucosa. | Self-monitoring |
| 13 | I can notice whether there are abnormal changes in the color or characteristics of my urine or stool. | Self-monitoring |
| 14 | I can monitor myself for signs of infection, such as fever, sore throat, or cough. | Self-monitoring |
| 15 | I can accurately describe my recent physical condition and discomfort to healthcare professionals. | Self-monitoring |
| 16 | When I feel anxious or depressed, I can regulate my emotions through relaxation, talking with others, or similar methods. | Emotional and daily life management |
| 17 | I can maintain a regular daily routine and try to ensure adequate sleep. | Emotional and daily life management |
| 18 | When physically able, I can maintain moderate activity or walking. | Emotional and daily life management |
| 19 | I can actively communicate with family members and friends about my illness and obtain their understanding and support. | Emotional and daily life management |
| 20 | I can face difficulties during treatment positively and maintain an optimistic attitude. | Emotional and daily life management |

Scoring method: Each item is scored from 0 to 10. Domain scores are calculated as the sum of item scores within each domain. The total score is calculated as the sum of all 20 items and ranges from 0 to 200, with higher scores indicating better self-management ability. No reverse-scored items were included.

Supplementary Table S2. Full item set of the study-specific nursing service satisfaction questionnaire

Instruction to participants: This questionnaire was used to assess patient satisfaction with nursing services during hospitalization and chemotherapy and was administered at the 3-month follow-up assessment. Participants were asked to rate each statement according to their actual experience on a scale from 0 (very dissatisfied) to 5 (very satisfied).

| No. | Item statement | Domain |
| --- | --- | --- |
| 1 | I was satisfied with how promptly nurses paid attention to changes in my condition. | Responsiveness |
| 2 | When I used the call bell, nurses came to me in a timely manner. | Responsiveness |
| 3 | Nurses responded to and handled my questions and needs promptly. | Responsiveness |
| 4 | Nurses explained my condition and nursing precautions in language that was easy to understand. | Communication effectiveness |
| 5 | Nurses listened patiently to my concerns without being dismissive. | Communication effectiveness |
| 6 | Nurses proactively informed me about chemotherapy-related side effects and how to cope with them. | Communication effectiveness |
| 7 | Nurses communicated with kindness and made me feel respected. | Communication effectiveness |
| 8 | I could feel that nurses understood and empathized with my pain and discomfort. | Perceived empathy |
| 9 | Nurses provided encouragement and psychological support during treatment. | Perceived empathy |
| 10 | Nurses noticed changes in my emotional state and took the initiative to ask about and comfort me. | Perceived empathy |
| 11 | Overall, I was satisfied with the nursing services provided during this hospitalization. | Overall evaluation |
| 12 | Overall, I would recommend the nursing services of this hospital to other patients. | Overall evaluation |

Scoring method: Each item is scored from 0 to 5. Domain scores are calculated as the sum of item scores within each domain. The total score is calculated as the sum of all 12 items and ranges from 0 to 60, with higher scores indicating greater satisfaction with nursing services. No reverse-scored items were included.
